# The Small Molecule Triclabendazole Decreases the Intracellular Level of Cyclic AMP and Increases Resistance to Stress in *Saccharomyces cerevisiae*

**DOI:** 10.1371/journal.pone.0064337

**Published:** 2013-05-08

**Authors:** Yong Joo Lee, Runhua Shi, Stephan N. Witt

**Affiliations:** 1 Department of Biochemistry and Molecular Biology, Louisiana State University Health Sciences Center at Shreveport, Shreveport, Louisiana, United States of America; 2 Feist-Weiller Cancer Center, Louisiana State University Health Sciences Center at Shreveport, Shreveport, Louisiana, United States of America; Kinki University School of Pharmaceutical Sciences, Japan

## Abstract

The Ras-adenylyl cyclase-protein kinase A nutrient-sensing pathway controls metabolism, proliferation and resistance to stress in *Saccharomyces cerevisiae*. The genetic disruption of this pathway increases resistance to a variety of stresses. We show here that the pharmacological inhibition of this pathway by the drug triclabendazole increases resistance to oxidants, heat stress and extends the chronological life. Evidence is presented that triclabendazole decreases the intracellular level of cyclic AMP by inhibiting adenylyl cyclase and triggers the parallel rapid translocation of the stress-resistance transcription factor Msn2 from the cytosol into the nucleus, as deduced from experiments employing a strain in which *MSN2* is replaced with *MSN2-GFP* (GFP, green fluorescent protein). Msn2 and Msn4 are responsible for activating the transcription of numerous genes that encode proteins that protect cells from stress. The results are consistent with triclabendazole either inhibiting the association of Ras with adenylyl cyclase or directly inhibiting adenylyl cyclase, which in turn triggers Msn2/4 to enter the nucleus and activate stress-responsible element gene expression.

## Introduction

The Ras – adenylyl cyclase – protein kinase A (PKA) nutrient-sensing pathway, which is controlled by glucose, regulates metabolism, cell division, entry into stationary phase and the stress response. The main components of this pathway are GTPases Ras1 and Ras2, adenylyl cyclase, which converts ATP to the second messenger cyclic AMP (cAMP) and pyrophosphate, the cAMP-dependent enzyme PKA, and the stress-resistance transcription factors Msn2 and Msn4. Ancillary components include phosphodiesterases that fine tune the level of the second messenger cAMP, and proteins that modulate the interactions of Ras with GTP and with adenylyl cyclase. The GTP-bound, activated form of membrane-associated Ras binds to membrane-associated adenylyl cyclase (encoded by *CYR1* and *CYR2*) which stimulates the latter to convert ATP to cAMP. cAMP diffuses into the cytosol where it binds to a regulatory subunit of PKA.

PKA is a hetero-tetramer composed of two catalytic subunits, which are encoded by three redundant genes (*TPK1*, *TPK2* and *TPK3*) in yeast, and two regulatory subunits, which are encoded by one gene (*BCY1*) [1], [2]. Bcy1 negatively regulates the catalytic subunits of PKA. During growth on abundant glucose, Ras stimulates adenylyl cyclase to synthesize cAMP, and the binding of cAMP to Bcy1 triggers it to dissociate from the catalytic subunits, which are then free to phosphorylate downstream effectors [3]. The free catalytic subunits are thought to hyper-phosphorylate the nuclear localization sequences of Msn2/4, which prevents them from entering the nucleus and activating STRE gene expression. PKA thus negatively regulates stress-responsible element gene expression. Put another way, abundant glucose leads to abundant intracellular cAMP, which turns off stress-responsible element gene expression, whereas low intracellular cAMP turns on gene expression.

Elegant genetic studies using yeast have shown that inactivation of the Ras – adenylyl cyclase – PKA pathway increases resistance to stress and extends the chronological life span [4], [5] (chronological life span, survival of a population of non-dividing cells) as well as the replicative life span [6] (replicative life span, number of daughter cells produced from one mother cell). A critical examination of the chronological aging and replicative aging techniques is given in [7].

We recently screened the Prestwick and NIH chemical libraries to identify drugs that protect *S. cerevisiae* from a unique form of cell death called sugar-induced cell death [8]. Sugar-induced cell death occurs when stationary-phase yeast cells are transferred into water with 2% glucose and no other nutrients [9]; cells die because of reactive oxygen species accumulation [10]. From approximately 1500 drugs we found two “hits” that partially protect cells from sugar-induced cell death – antimycin A and 5-chloro-6-(2,3-dichlorophenoxy)-2-(methylthio)-1*H*-benzimid-azole (triclabendazole). Antimycin A is a mitochondrial complex II poison, and triclabendazole is an antihelminthic drug that is used to treat liver flukes in livestock and man. We found that triclabendazole protects yeast cells from death induced by the Parkinson's disease-related protein alpha-synuclein (α-syn), which trigger the accumulation of reactive oxygen species [11], and rat PC12 cells from hydrogen peroxide-induced cell death [12]. Herein, we used yeast to probe the mechanism by which triclabendazole protects cells from various stresses.

## Results

### Triclabendazole increases resistance to stress

**Figure 1:**
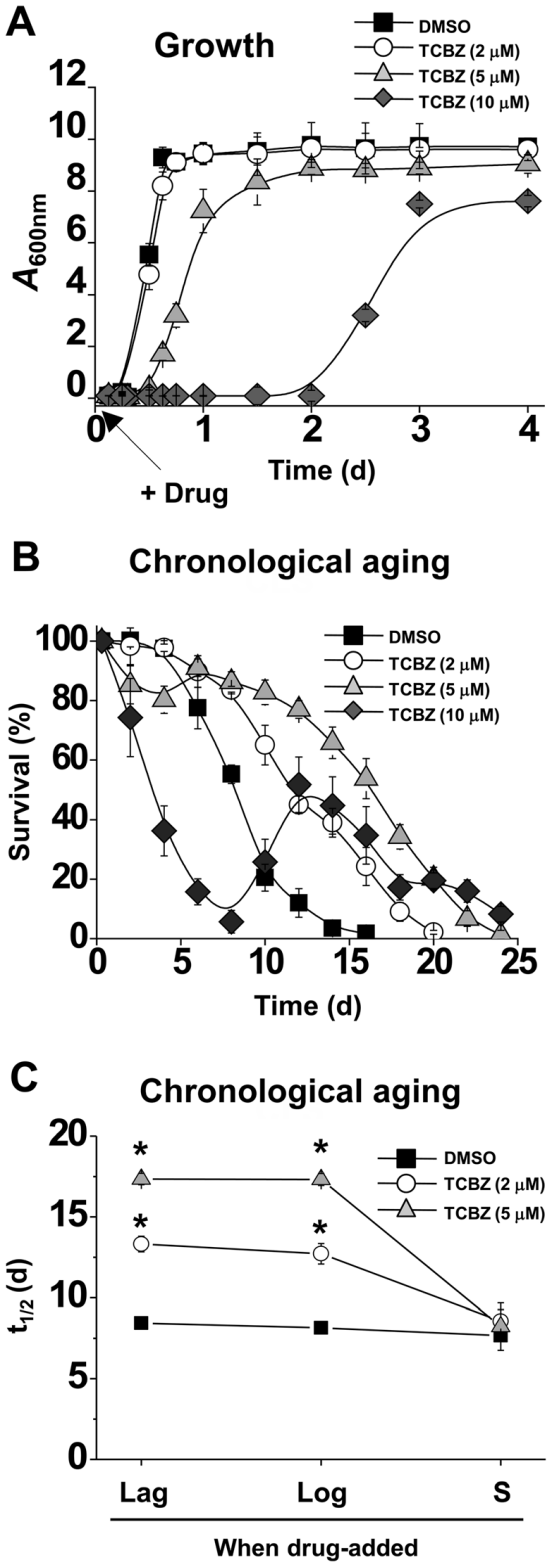
Triclabendazole extends the yeast chronological life span. (A) Growth with triclabendazole or vehicle (DMSO, 0.1%). Drug was added during lag-phase (arrow). Values are the mean ± standard deviation (SD) of the three independent experiments. (B) Chronological aging assay (survival as a function of time). Drug was added during lag-phase, and then cells were pre-grown for 48 h ( =  day 0 of life span assay) (2, 5 μM DMSO and triclabendazole, TCBZ) or for 72 h ( =  day 0 of life span assay) (10 μM triclabendazole). Viability was measured as a function of time. Values are the mean ± SD of the four independent experiments. (C) Plot of life span *versus* phase when drug was added. Life span values (*t_1/2_*) are the mean ± SD of the four independent experiments. *, *p*<0.001 (versus DMSO).

**Figure 2:**
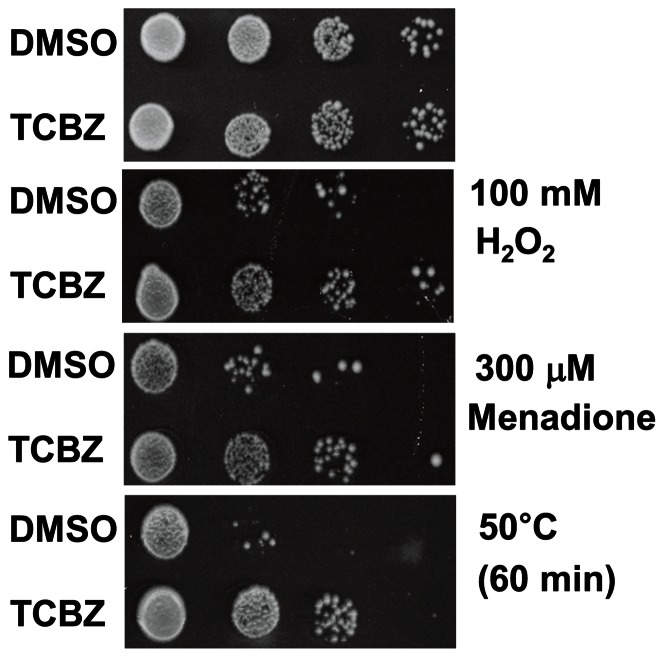
Triclabendazole protects cells from oxidative and heat stresses. Triclabendazole (5 μM) or vehicle was added to diluted cells in SC-glucose medium, and then cells were incubated at 30°C with shaking for 4 days. Cells were removed from the culture, serially diluted in PBS, incubated with oxidant or heated and spotted onto YPD plates. Plates were incubated at 30°C for 3 days.

The effects of triclabendazole on growth, survival and response to various stresses were determined. Cells were inoculated into liquid medium containing triclabendazole or vehicle (DMSO) and the absorbance of the culture was monitored over several days (Fig. 1A). 2 μM triclabendazole had no effect on growth compared to control cells with vehicle, whereas increasing growth inhibition occurred at 5 and 10 μM, and 20 μM killed cells. For the chronological aging assay, cells were inoculated into liquid medium containing triclabendazole or vehicle, cultures were incubated with shaking for 2 or 3 days, and then an experiment was started. The mean life span of wild-type cells with vehicle (8.2±0.2 d) increased by 62% (13.3±0.5 d) and 111% (17.3±0.4 d) upon treatment with 2 μM and 5 μM triclabendazole (Fig. 1 B), respectively. At 10 μM triclabendazole, the life span could not be determined because the decay is complicated by an adaptive re-growth pattern [13], which is also known as ‘gasping’ [7]. To be effective, 2 to 5 μM triclabendazole had to be added during lag- or log-phase but not during stationary-phase (Fig. 1 C). Triclabendazole also protected cells from oxidants (hydrogen peroxide and menadione) and heat stress (Fig. 2).

One explanation for the adaptive re-growth (Fig. 1B) is that high dose triclabendazole is mutagenic. To address this possibility, we examined the spontaneous mutation rates of cells treated with different concentrations of triclabendazole using the canavanine assay [14]. Canavanine, which is an arginine analog, is toxic to yeast. Mutations that prevent canavanine from being taken up by yeast cells will result in mutants that can grow in media containing canavanine. Over the 14-day period, the mutational rates for DMSO- and 5 μM triclabendazole-treated cells were identical, each mutational rate approximately doubled over the 14-day time frame (Fig. S1). In contrast, for cells treated with 10 μM triclabendazole the mutational rate was slightly higher, tripling over the same time period. We typically used 5 μM triclabendazole for the experiments in this study.

**Figure 3:**
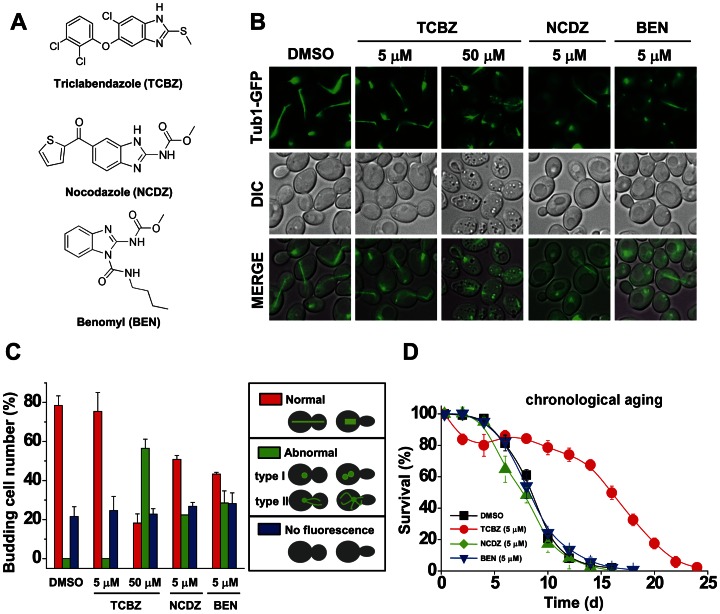
Triclabendazole-induced increase in life span is not due to microtubule destabilization. (A) Triclabendazole, nocodazole and benomyl. (B) Spindle formation. Cells expressing Tub1-GFP were pre-grown in SC-glucose until mid-log phase, incubated with the indicated drug for 1 h, and then imaged by fluorescence microscopy. (C) Plot of cells exhibiting different spindle structures. Each value was obtained from three independent experiments, where the total number of cells counted was 200–300. Error bars are ± SD. S, stationary phase. (D) Chronological aging assay. The indicated drug was added in the lag-phase, cells were pre-grown for 48 h ( =  day 0 of life span assay), and viability was measured as a function of time. Values are the mean ± SD of the four independent experiments.

In vivo studies have indicated that triclabendazole inhibits β-tubulin from the liver fluke (*Fasciola hepatica*) [15], although no definitive binding site for triclabendazole or its metabolites on β-tubulin has ever been identified. The related drug thiabendazole (Table S1) depolymerizes microtubules in *Schizosaccharomyces pombe* cells [16], although a high concentration (500 μM) is required. We tested whether triclabendazole inhibits tubulin polymerization in a yeast strain expressing an α-tubulin-green fluorescent protein fusion (Tub1-GFP). α-Tubulin and β-tubulin form heterodimers that polymerize into microtubules. Benomyl and nocodazole (Fig. 3A) [17], which disrupt microtubules, were used as controls. Using fluorescence microscopy to visualize mitotic spindles, which are composed of microtubules, we found that 5 μM triclabendazole had no adverse effect on spindle morphology, whereas the same concentration of benomyl or nocodazole caused aberrantly shaped spindles (Fig. 3B, C), which is consistent with these latter two drugs disrupting the polymerization of tubulin. On the other hand, at a 10-fold higher concentration (50 μM), triclabendazole clearly disrupted spindles. We also found that in the chronological aging assay, wild-type cells treated with vehicle or 5 μM benomyl or nocodazole had the same life span, whereas treating cells with 5 μM triclabendazole boosted life span by over 100 % compared to untreated cells (Fig. 3D). Thus, triclabendazole at 5 μM extends the chronological life span of yeast cells without disrupting mitotic spindles. Collectively, at high concentrations (>10 μM) triclabendazole disrupts microtubules and is toxic, whereas at lower concentrations (2-5 μM) the drug does not disrupt microtubules and is cytoprotective.

### Triclabendazole and rapamycin – effects on carbon source utilization and O_2_ consumption

**Figure 4:**
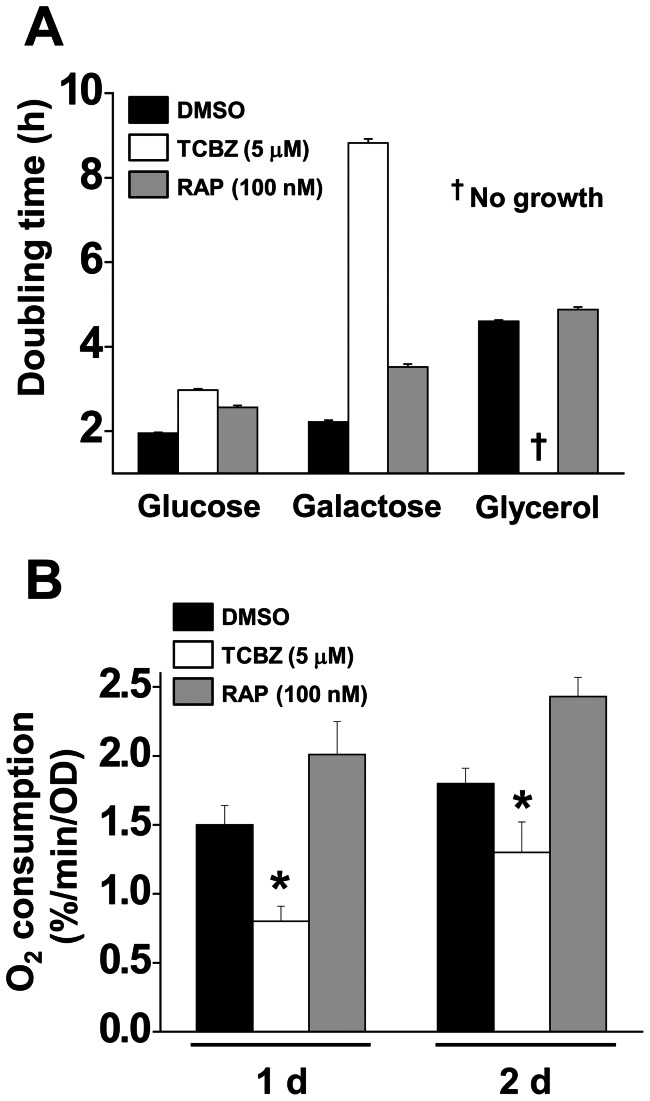
Effects of triclabendazole and rapamycin on carbon source utilization and O_2_ consumption. (A) Carbon source utilization. Cells were inoculated in SC-glucose, -galactose, or -glycerol medium with the drug (triclabendazole, TCBZ; rapamycin, Rap) or DMSO and incubated with shaking at 30°C. Absorbance (*A*_600 nm_) was measured every 3 h, and doubling time was calculated as described in the methods. Values are the mean ± SD of the three independent experiments. (B) O_2_ consumption. Cells were inoculated in SC-glucose medium with the drug or DMSO and incubated for indicated times at 30°C with shaking. Before determining the rate of O_2_ consumption, the absorbance of each sample was measured. Samples were then transferred to an airtight chamber maintained at 30°C, and the oxygen content was monitored for at least 5 min. Values are the mean ± SD of the three independent experiments. *, p<0.05 (versus DMSO).

Rapamycin, which is a pharmacological inhibitor of the TOR pathway in yeast, extends the yeast chronological life span [18], [19]. We were curious to compare carbon source utilization and O_2_ consumption in cells treated with triclabendazole or rapamycin. Fig. 4A shows that cells treated with triclabendazole (5 μM) grew normally in glucose medium but displayed a severe growth defect in galactose and no growth in glycerol (Fig. 4A). These results are in contrast to control cells (DMSO) and cells treated with rapamycin (100 nM), which grew on the each of the three carbon sources. Fig. 4B shows O_2_ consumption data for cells treated with vehicle, triclabendazole or rapamycin. Triclabendazole and rapamycin had opposite effects on O_2_ consumption, in that triclabendazole inhibited O_2_ consumption whereas rapamycin stimulated it, in agreement with a previous report [20]. Cells treated with triclabendazole and rapamycin exhibit very different carbon source utilizations and very different O_2_ consumption profiles, which indicates that these two drugs affect different cellular pathways.

### The protective effect of triclabendazole depends on the transcription factors Msn2/4

**Figure 5:**
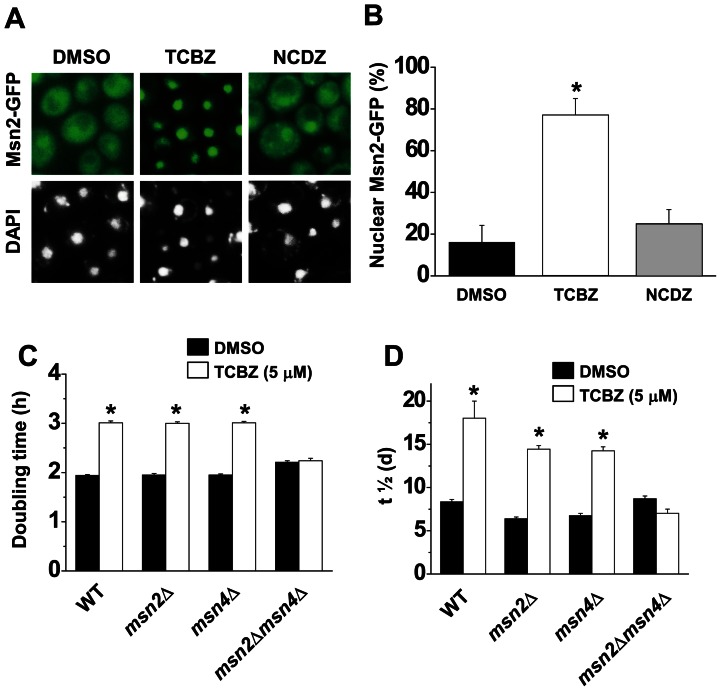
Triclabendazole activates Msn2/4. (A) Triclabendazole activates nuclear localization of the Msn2. Msn2-GFP were pre-grown to A_600_  = 0.5–0.6, the indicated drug was added for 2 h, and cells were then visualized by fluorescence microscopy. Nuclei were visualized using DAPI. (B) Percent of cells with nuclear Msn2-GFP. Values were obtained from four independent experiments; the total number of cells counted was 300–350. Error bars are ± SD. *, *p*<0.001 (versus DMSO or nocodazole). (C) Doubling time. Indicated cells were inoculated in SC-glucose medium with triclabendazole or DMSO and incubated with shaking at 30°C. Absorbance (*A*_600 nm_) was measured every 3 h, and doubling time was calculated as described in the methods. Values are the mean ± SD of the three independent experiments. *, *p*<0.001 (versus DMSO), (D) Plot of mean life span (*t*_1/2_) values obtained from chronological aging assay of *msn2*Δ, *msn4*Δ, *msn2*Δ*msn4*Δ and wild-type cells. Values are the mean ± SD of the three independent experiments. *, *p*<0.001 (versus DMSO). TCBZ, triclabendazole; NCDZ, nocodazole.

Because triclabendazole must be added during lag- or log-phase but not stationary-phase (Fig. 1C), we surmise that triclabendazole up-regulates genes that protect cells from oxidative stress. The response to oxidative stress in yeast is mainly controlled by the two non-essential and partially redundant stress-resistance transcription factors Msn2 and Msn4. Under non-stress conditions these two transcription factors localize to the cytosol, whereas in response to various stresses they redistribute into the nucleus and activate transcription. We found that triclabendazole triggered the rapid redistribution of Msn2 from the cytosol into the nucleus in exponential-phase cells, as illustrated by a strain with an integrated *MSN2-GFP* allele that was incubated with triclabendazole and then imaged by fluorescence microscopy (Fig. 5 A). The compound 4′, 6-diamidino-2-phenylindole dihydrochloride (DAPI) in conjunction with fluorescence microscopy was used to visualize the nucleus. The cellular response to triclabendazole was robust in that it induced a 358% increase in the nuclear localization of Msn2-GFP compared to the same cells without the drug or with nocodazole (Fig. 5 B). We next asked whether Msn2/4 are required for the pro-survival effects of triclabendazole. Doubling time and the chronological aging assay were used. Figs. 5C, D show that triclabendazole loses it biological effect on yeast cells only when Msn2 and Msn4 are both deleted. The results show that triclabendazole triggers the rapid movement of Msn2 into the nucleus, and the protective effect of this drug is abolished upon deletion of *MSN2/4*.

### Triclabendazole decreases the intracellular level of cAMP

**Figure 6:**
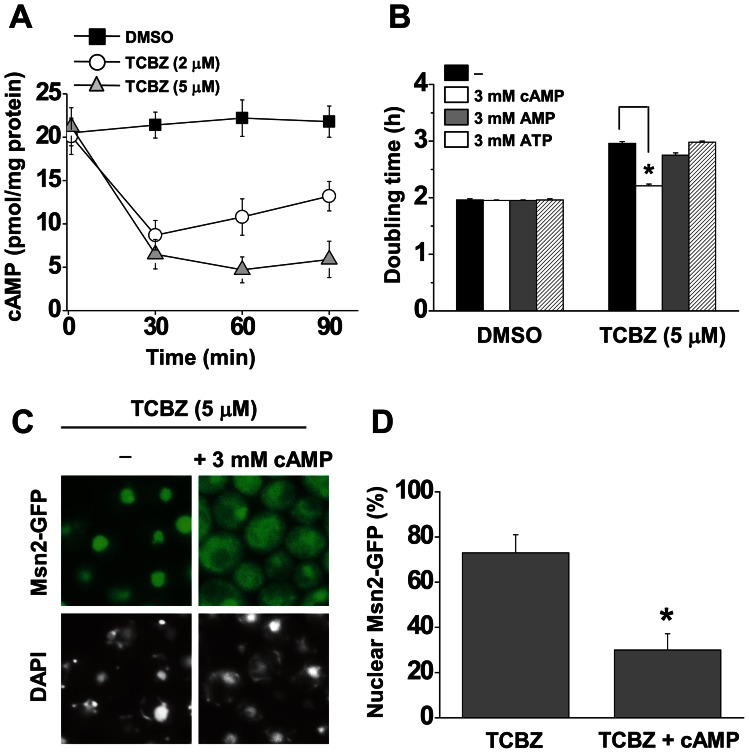
Triclabendazole decreases intracellular cAMP. (A) Intracellular level of cAMP. Cells (wild-type) were inoculated in SC-glucose medium and grown to mid-log phase, drug was added, and the samples were incubated for the indicated times. cAMP was extracted from cells and quantified using an immunoassay (Cell Signaling). Values are the mean ± SD of the four independent experiments. (B) Doubling time. Cells (wild-type) were inoculated into SC-glucose containing triclabendazole (5 μM) or DMSO with added nucleotide (cAMP, AMP or ATP) and incubated with shaking at 30°C. Doubling times are the mean ± SD of the three independent experiments. *, *p*<0.005. (C) Msn2-GFP localization. Cells expressing Msn2-GFP were grown to mid-log phase, and then triclabendazole (5 μM) or triclabendazole (5 μM) and cAMP (3 mM) was added. The cells were imaged by fluorescence microscopy after 2 h. (D) Percent of cells with nuclear-localized Msn2-GFP. Values are means ± SD from two independent experiments, where the total number of cells counted was 200–300. *, *p*<0.005.

On the basis of the above findings, we asked whether triclabendazole alters the intracellular level of cAMP in yeast cells. cAMP was detected in yeast cell lysates using an enzyme immunoassay, which has been used before to detect cAMP in yeast (see Materials and Methods) [21]. Triclabendazole decreased the intracellular content of cAMP in wild-type cells by 70% within 30 min after adding the drug (to exponential-phase wild-type cells) (Fig. 6A), and the decrease was still evident after 90 min. Whether cAMP added in the liquid medium would reverse the effect of triclabendazole on the yeast doubling time and on the relocalization of Msn2-GFP into the nucleus was also examined. Yeast cells can take up cAMP from the cell culture medium [22]. Added cAMP (3 mM), but not added AMP or ATP, reversed the triclabendazole-induced increase in doubling time (Fig. 6B) and also blocked the triclabendazole-induced relocalization of Msn2-GFP into the nucleus. Specifically, 73% of triclabendazole-treated cells displayed Msn2-GFP in the nucleus; whereas, in the same cells incubated with triclabendazole (5 μM) and cAMP (3 mM) this value decreased to 30% (Fig. 6 C, D).

Triclabendazole is a benzimidazole. Four other antihelminthic benzimidazoles – albendazole, fenbendazole, mebendazole and thiabendazole – as well as compounds structurally related to triclabendazole were tested in the various assays (Table S1). Fenbendazole was the only compound with activity similar to triclabendazole (Fig. S2A). Fenbendazole induced the redistribution of Msn2-GFP from the cytosol into the nucleus (Fig. S2 B, C) and decreased the intracellular level of cAMP in yeast cells (Fig. S2D). Triclabendazole has two metabolites that could have biological activity. One is a sulfoxide (-SO) and the other is a sulfone (−SO_2_). These two metabolites extended the yeast chronological life span (Table S2), although 4- to 10-fold higher concentrations were required compared to the parent drug.

### The protective effect of triclabendazole depends on Rim15

**Figure 7:**
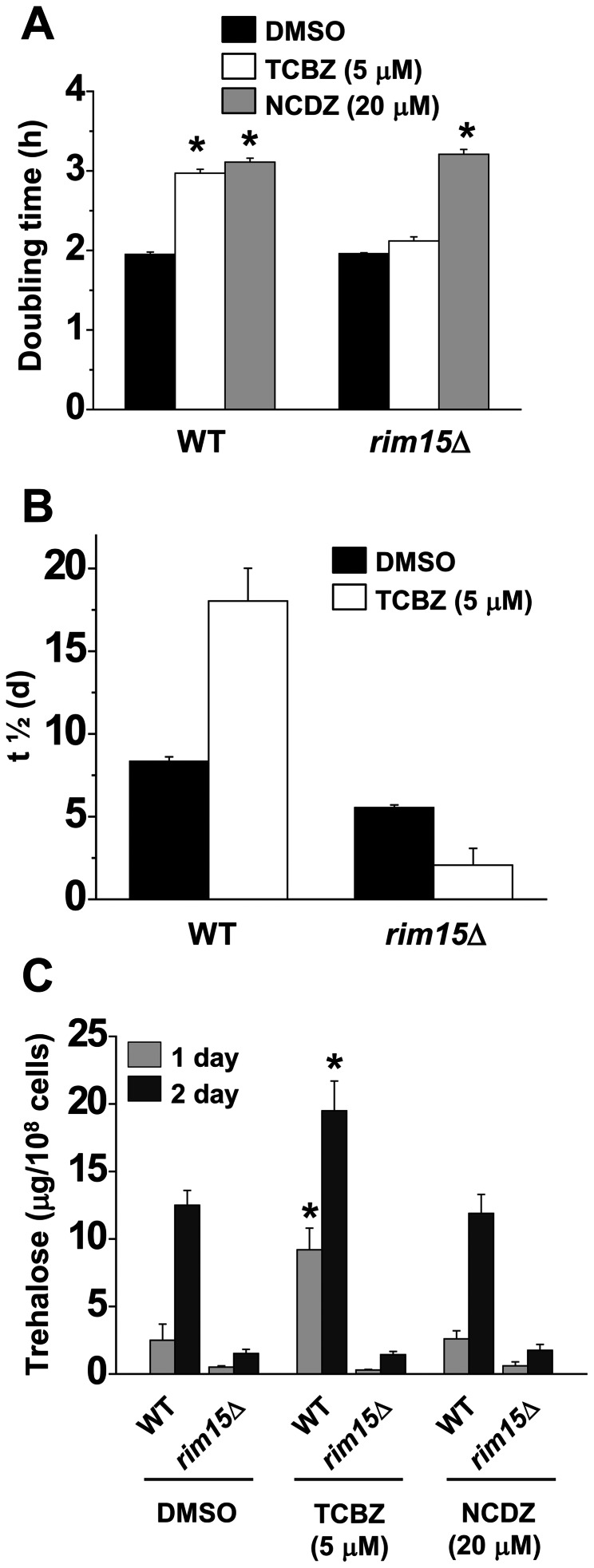
Rim15 is required for triclabendazole biological activity. (A) Doubling time. Cells (wild-type, WT, or *rim15*Δ) were inoculated in SC-glucose medium with the indicated drug or DMSO and incubated at 30°C with shaking. Doubling time values are the mean ± SD of the three independent experiments. *, *p*<0.001 (versus DMSO). (B) Chronological aging. Cells were inoculated in SC-glucose medium with drug or DMSO and incubated at 30°C with shaking for 48 h ( =  zero point for survival curve). Survival curves give the percent of viable cells over time. Values are the mean ± SD of the three independent experiments. (C) Trehalose accumulation. Cells were inoculated in SC-glucose medium with drug or DMSO, incubated at 30°C, and at the indicated time the trehalose content was determined. Values are the mean ± SD of the three independent experiments. *, *p*<0.01 (versus DMSO). Triclabendazole, TCBZ; nocodazole, NCDZ.

Rim15 controls entry into stationary phase [23] and is negatively regulated by PKA [24]. *rim15*Δ cells were tested in several assays to determine whether Rim15 is a component of the pathway modulated by triclabendazole. Figure 7 shows the effects of triclabendazole and nocodazole on growth, life span, and trehalose content of wild-type and *rim15*Δ cells. (i) Triclabendazole (5 μM) and nocodazole (20 μM) increased the doubling time of wild-type cells by ∼50% compared to untreated cells (Fig. 7A). In contrast, triclabendazole failed to affect the doubling time of *rim15*Δ cells, whereas nocodazole increased the doubling time of *rim15*Δ cells by ∼50% compared to untreated cells. (ii) Triclabendazole extended the chronological life span (t_1/2_) of wild-type cells but not of *rim15*Δ cells (Fig. 7B). (iii) In the trehalose assay, the indicated drug was added to diluted cells, and trehalose content was determined after 1 d and 2 d of growth. Triclabendazole, but not nocodazole, increased the amount of trehalose in wild-type cells compared to the same cells with vehicle (Fig. 7C). Whether treated with DMSO, triclabendazole or nocodazole, *rim15*Δ cells exhibited the same low levels of trehalose over the 2 days. The results show that Rim15 is required for the biological activity of triclabendazole, and similar results were obtained with a *bcy1*Δ*bcy1*Δ mutant (Fig. S3). The combined results show that Msn2/4, Rim15 and Bcy1 are required for the protective effect of triclabendazole.

Triclabendazole could decrease the intracellular level of cAMP in several ways (see Discussion). The simplest way is that triclabendazole inhibits adenylyl cyclase. Less likely is that triclabendazole activates a phosphodiesterase, which then rids cells of cAMP. Each possibility was explored.

### Triclabendazole does not stimulate the phosphodiesterase Pde2

**Figure 8:**
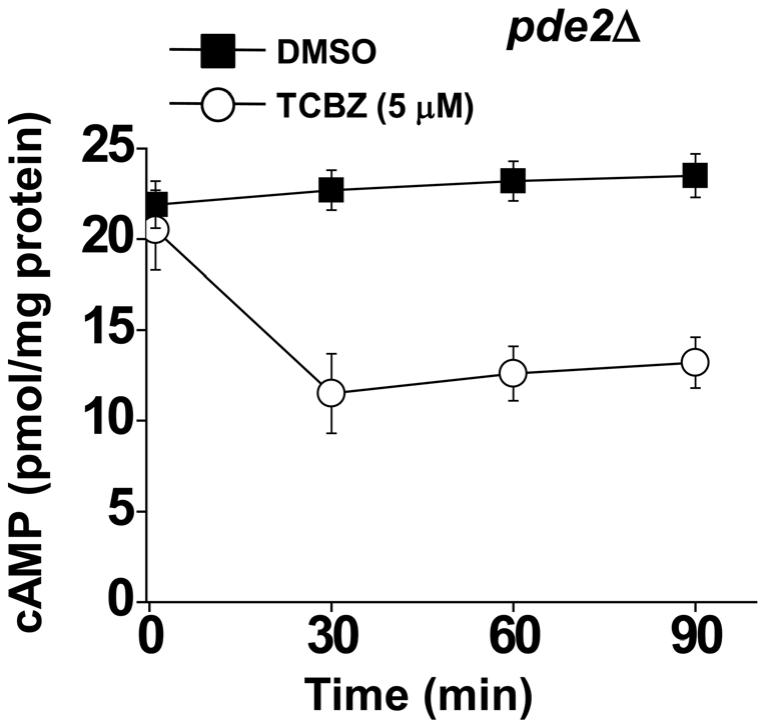
Triclabendazole decreases cAMP in *pde2*Δ cells. Intracellular level of cAMP in *pde2*Δ cells. Cells (*pde2*Δ) were inoculated in SC-glucose medium and grown to mid-log phase, drug was added, and the samples were incubated for the indicated times. cAMP was extracted from cells and quantified using an immunoassay (Cell Signaling). Values are the mean ± SD of the four independent experiments.

Phosphodiesterases catalyze the conversion of cAMP to AMP, and *S. cerevisiae* express two phosphodiesterases, Pde1 and Pde2, which are low- and high-affinity cAMP phosphodiesterases, respectively. One possibility is that triclabendazole is an allosteric activator of Pde2. A drug that allosterically activates a phosphodiesterase would increase the activity of the enzyme, causing more conversion of cAMP to AMP. If triclabendazole allosterically activates Pde2, triclabendazole should fail to decrease cAMP in a *pde2*Δ deletion strain. To this end, we found that triclabendazole (5 μM) decreased the level of cAMP by 50% in *pde2*Δ cells compared to the same cells with vehicle (Fig. 8). These findings show that triclabendazole does not activate Pde2.

### Possible inhibition of adenylyl cyclase by triclabendazole and fenbendazole

To determine whether triclabendazole inhibits adenylyl cyclase (Cyr1), we prepared a yeast plasma membrane extract and examined the ability of Cyr1 in this extract to synthesize cAMP with and without added drug. The drug of interest was added to the extract, and then GppNHp, which stimulates Cyr1 to synthesize cAMP from ATP, was added. 2,5-dideoxyadenosine (2,5-DDA) [25], which is a commercially available, cell-permeable inhibitor of human adenylyl cyclase, was used as a control. Cyr1 activity was reported as pmol cAMP/mg protein/min at 30°C. Triclabendazole and fenbendazole each decreased Cyr1 activity in a dose-dependent manner, yielding a 70% reduction in activity at 1 μM (Table 2). In contrast, at 1 μM neither ALBZ nor 2, 5-DDA affected Cyr1 activity, whereas at a much higher concentration (300 μM), 2, 5-DDA decreased Cyr1 activity by 10%. The findings support two possibilities: Triclabendazole and fenbendazole directly inhibit Cyr1, or they block the interaction of GTP-Ras with Cyr1 – either mode of action would prevent the synthesis of cAMP.

**Table 2:** Adenylyl cyclase activity in a plasma membrane extract.

Drug	Concentration (μM)	Adenylyl cyclase activity (pmol of cAMP/mg/min)^a^	% Change*^b^*
DMSO	−	40.6±5.6	−
Triclabendazole	0.1	25.2±6.2	−37.9
	1	12.3±4.7	−69.6
Fenbendazole	0.1	22.2±3.8	−45.3
	1	14. 2±5.9	−64.9
Albendazole	0.1	40.6±5.3	−0.2
	1	41.2±3.1	+1.0
	300	40.6±5.7	−0.1
2,5-DDA	0.1	41.7±3.6	+2.6
	1	41.4±2.7	+2.0
	300	34.6±2.4	−9.8

a Adenylyl cyclase activity in a yeast plasma membrane extract was measured in the presence of the triclabendazole, fenbendazole, albendazole, or 2,5-dideoxyadenosine (2,5-DDA) as described in the “Materials and Methods”. Values are the mean ± SD of the three independent experiments. *^b^* The percentage change was determined by comparing the experimental treatments triclabendazole, fenbendazole, and 2,5-DDA to DMSO.

## Discussion

**Figure 9:**
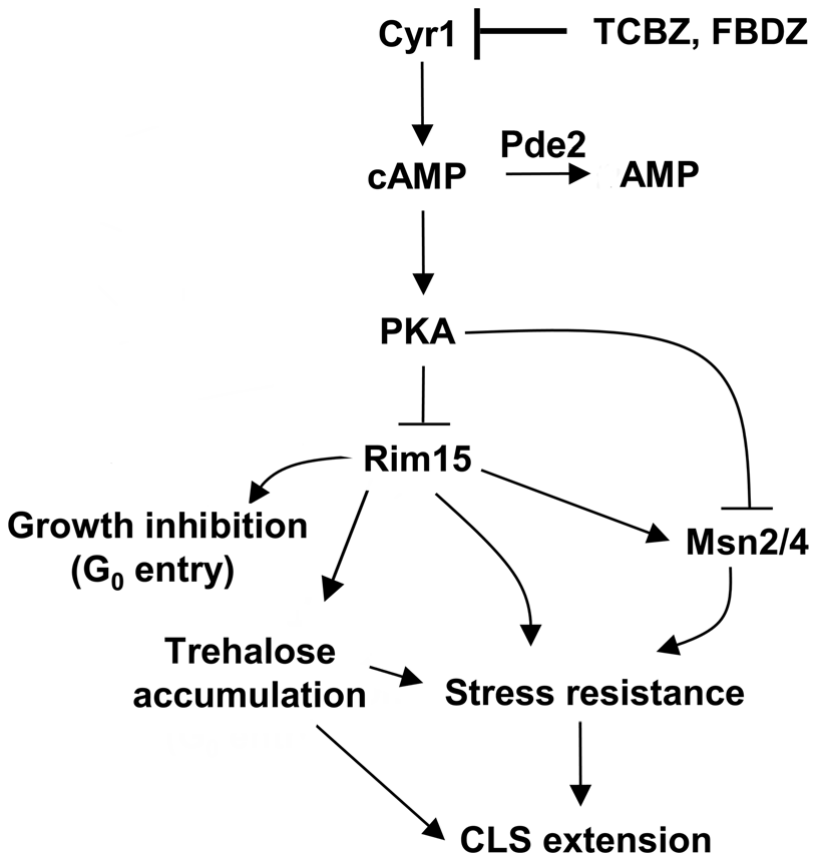
Model for the mechanism of action of triclabendazole. Triclabendazole (TCBZ) and fenbendazole (FBDZ) inhibit adenylyl cyclase (Cyr1), which decreases the intracellular level of cAMP. Low levels of cAMP maintain PKA in an inactive state, which results in the activation of Msn2/4. Msn2/4 trigger the transcription of stress-responsive genes. The accumulation of trehalose can help cells survive in stationary phase as well as provide protection from proteotoxic stress. CLS, chronological life span.

We found that the two antihelminthic drugs, triclabendazole and fenbendazole, inhibit the synthesis of cAMP, and the decreased level of cAMP activates the stress-resistance transcription factors Msn2/4. Our findings regarding the protective effect of these two drugs can be explained by the model shown in Figure 9.

### Fluke diseases are a worldwide health problem

Diseases caused by trematodes (flukes) are a major worldwide health problem. Several comprehensive reviews are available (see [26], [27]). These infections, which afflict livestock and humans, target the liver, lung, and intestines and are obtained from consuming unwashed vegetables and from drinking contaminated water. Over 200 million people are at risk for fluke infections, and currently it is estimated that 2.5 million people suffer from these infections. Triclabendazole has been used since 1990 for the treatment of fluke infections, and typically one dose eliminates the infection [28]. The drug is registered for humans in four countries – Egypt, Ecuador, Venezuela and France – and it has been used in many other countries [29]. The World Health Organization placed triclabendazole on its list of essential drugs in 1997. There are a considerable number of case studies of triclabendazole and much is known about its pharmacodynamics and toxicity in humans. Overall, triclabendazole is well tolerated by humans at doses of 10–12 mg/kg, and adverse events are negligible. The mechanism of action of triclabendazole and its two metabolites has not been determined, although various reports have indicated that triclabendazole and its metabolites inhibit tubulin, protein synthesis, and RNA synthesis in the flukes [26]. We found that concentrations of triclabendazole greater than 10 μM severely inhibit growth, disrupt microtubules, which are composed of tubulin, and kill yeast cells (Figs. 1A, 3; Tables S1, S2). Thus, our results that triclabendazole disrupts tubulin in yeast is similar to reports that the drug disrupts tubulin in flukes.

Triclabendazole also has pro-survival effects on cells. With its amazing toolbox, yeast may be the perfect organism to dissect the mechanisms by which triclabendazole can protect or harm. Below we discuss aspects of how triclabendazole protects yeast cells from stress.

### Triclabendazole phenocopies *cyr1* and *ras* mutants – the protective effect of triclabendazole

Genetically disrupting the Ras-adenylyl cyclase-PKA pathway yields a phenotype that is remarkably similar to what we found when cells are treated with triclabendazole or fenbendazole. For example, one study used transposon-mutagenized yeast cells to identify long-lived mutants and found that mutations in *CYR1* or *SCH9* extend the chronological life span up to three-fold compared to wild-type cells [4]. Strikingly, the stress-resistance transcription factors Msn2/4 and the Rim15 kinase are required for the life span extension of these mutants, which is identical to what we found vis-à-vis the triclabendazole-mediated extension of the chronological life span. That is, deleting *MSN2* and *MSN4* or *RIM15* or *BCY1* abolished the ability of triclabendazole to extend the chronological life span (Figs. 5, 7, S3). Genetic disruption of the Ras-adenylyl cyclase-PKA pathway increases resistance to stress, as evidenced by the *cyr1::nTN* mutant being resistant to heat shock, hydrogen peroxide and menadione [4]. Pharmacologically inhibiting this pathway should also promote resistance to various stresses, and indeed triclabendazole increased cellular resistance to heat, hydrogen peroxide and menadione (Fig. 2).

The deletion of *RAS2*, which encodes for the small GTP-binding protein Ras2, also significantly extends the yeast chronological life span [4]. Ras proteins are like molecular switches: the GTP bound form is active, whereas the GDP bound form is inactive. Ras2 localizes to the inner leaflet of the plasma membrane via a farnesyl and palmitoyl groups that are covalently attached to its carboxyl-terminus [30]. In the plasma membrane, the GTP-bound Ras2 associates with Cyr1 and stimulates Cyr1 to synthesize cAMP from ATP [31]. The chronological life span extension occurs in *ras2*Δ cells because Cyr1 cannot synthesize sufficient amounts of cAMP in the absence of Ras2. The decreased global level of cAMP triggers Msn2/Msn4 to transcribe genes that encode for protective proteins (Fig. 9). Note that cells with mutated *RAS2* also have severe growth defects when grown in media with non-fermentable carbon sources such as acetate and glycerol and increased accumulation of glycogen [32], [33]. We found that, compared to untreated control cells, triclabendazole-treated cells exhibited decreased O_2_ consumption (Fig. 4B), accumulated trehalose (Fig. 7C), and had difficulty utilizing galactose and glycerol (Fig. 4A). Triclabendazole-treated cells exhibit a complex phenotype that is strikingly similar to the phenotypes exhibited by *cyr1* and *ras* mutants.

### Triclabendazole – mechanism of protection

Although the triclabendazole-induced decrease in the intracellular level of cAMP is best explained by triclabendazole inhibiting adenylyl cyclase, two other possibilities were considered. First, triclabendazole and fenbendazole could decrease the level of cAMP by allosterically activating the phosphodiesterase Pde2. However, because triclabendazole decreased the intracellular level of cAMP in *pde2*Δ cells compared to control cells (Fig. 8), this possibility was ruled out. Second, triclabendazole could inhibit the binding of GTP to Ras. Such inhibition would result in a failure of Ras to activate Cyr1 and a concomitant decrease in cAMP. If triclabendazole competitively inhibits the binding of GTP to Ras, then the fraction of Ras molecules bound with triclabendazole, *f*_Ras-TCBZ_, is (eq. 1):
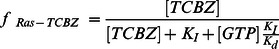
(1)where [TCBZ] and [GTP] are the intracellular concentrations of triclabendazole and GTP and *K_d_* and *K_I_* are the equilibrium dissociation constants for GTP and triclabendazole from Ras, respectively. Because GTP binds to small GTPases like Ras with picomolar dissociation constants [34], we suggest that *K_d_* ≈ 100×10^−12^ M, and because triclabendazole inhibits cAMP production in the membrane fraction by 50% at 1 μM, we estimate that *K_I_* ≈1×10^−6^ M. Using these values for *K_d_* and *K_I_* and the values of 5 μM triclabendazole and 1.5 mM GTP [35] in eq. 1, the fraction of Ras molecules bound with triclabendazole is 3×10^−7^, which is essentially zero. The available evidence indicates that triclabendazole and fenbendazole either block GTP-Ras-Cyr1 complex formation or directly inhibit adenylyl cyclase/Cyr1. Either mode of action would decrease the level of cAMP.

Several triclabendazole analogs and metabolites were tested in this study. The four triclabendazole analogs – albendazole, fenbendazole, mebendazole and thiabendazole–are used to treat liver fluke infections in humans and livestock, but only fenbendazole was active in our assays. Fenbendazole and triclabendazole exhibited nearly identical activities in the various assays (Fig. S2; Tables 2, S1). The two metabolites of triclabendazole, the sulfoxide and the sulfone, showed no activity at 5 μM, whereas at higher concentrations each significantly extended the chronological life span (Table S2). For example, at 20 μM, the sulfoxide (triclabendazole-SO) extends the chronological life span by 80% compared to untreated cells, whereas triclabendazole is cytotoxic at this concentration. At 50 μM, the sulfone (triclabendazole-SO_2_) extends the chronological life span by 64% compared to untreated cells, whereas triclabendazole and the sulfoxide are cytotoxic. Assuming that triclabendazole and its two metabolites enter yeast cells with the same efficiency, then triclabendazole is the most potent drug because it exerts it beneficial effects at only 5 μM whereas a 4–10-fold higher concentration of the two metabolites was needed for the same beneficial effects.

Yeast adenylyl cyclase is different from mammalian adenylyl cyclases in terms of structure and regulation. For instance, Ras proteins in yeast associate with and regulate the activity of Cyr1 [36], whereas in mammalian cells G-protein coupled receptors and other factors (forskolin, phorbol esters, metals) associate with and regulate the activity of adenylyl cyclases [37]. Many drugs decrease the level of cAMP, although they typically function upstream of the cyclase. In recent years more effort has been placed into designing small molecule inhibitors of adenylyl cyclases, but this effort is complicated by the fact that humans have ten adenylyl cyclase isoforms. There are two types of adenosine-dependent sites for inhibitors on the adenylyl cyclases: molecules that bind to the “R-site” must contain a ribose moiety, whereas molecules that bind to the “P-site” must have a purine ring [38], although a P-site inhibitor without a purine ring was recently reported [39]. Inhibitors of the “P-site” have dissociation constants in the micromolar range (1–300 μM) [39], [40], which is the general range in which triclabendazole inhibits cAMP production in yeast. Triclabendazole but not albendazole also protects rat PC12 cells from hydrogen peroxide-induced cell death. Perhaps triclabendazole is a P-site inhibitor. Clearly, further experiments are needed to determine whether triclabendazole and fenbendazole inhibit mammalian adenylyl cyclases.

### cAMP, alpha-synuclein and Parkinson's disease (PD)

We recently reported that triclabendazole extends the chronological life span of yeast cells expressing the PD-associated protein alpha-synuclein (α-syn) by 64% compared to untreated cells [12]. It is noteworthy that in an unbiased screen of a yeast genomic library it was discovered that three genes – *YPK9*, *CDC5*, and *PDE2* – individually, in high copy protect against human α-syn in yeast and also in worm, fly and rat cells [41]. Thus, decreasing cAMP by a drug (triclabendazole or fenbendazole) or by overexpressed phosphodiesterase (*PDE2*) protects against α-syn in model organisms. Lastly, dopamine receptors are G-protein coupled receptors, and given that these receptors are involved in such a multitude of processes – learning, motor control, memory, pleasure and prolactin release – there are several subtypes of these receptors. The D_1_ family of dopamine receptors stimulates adenylyl cyclase to synthesize cAMP, whereas the D2 family inhibits the enzyme and thus decreases the intracellular level of cAMP [42]. Whether reducing cAMP in selected neurons can be neuroprotective in PD must await further experimentation.

## Materials and Methods

### Cells, media and reagents

Yeast strains used in this study are listed in Table 1. Liquid rich medium (YPD) contained 1% (w/v) yeast extract, 2% (w/v) peptone and 2% (w/v) dextrose; YPD for plates also contained 2% bacto-agar (BD, Franklin Lakes, NJ). Synthetic complete (SC) medium contained 0.67% (w/v) yeast nitrogen base, 0.16% (w/v) yeast drop-out mix (- Leu), 0.02% L-leucine (Sigma-Aldrich, St-Louis, MO) and 2% (w/v) glucose. Cultures in liquid media were grown with shaking at 30°C.

**Table 1:** Strains used in this study.

Strain	Description	Source
WT (BY4741)	*MAT*a *his3*Δ*1 leu2*Δ*0 met15*Δ*0 ura3*Δ*0*	ATCC (Manassas, VA)
WT (BY4742)	*MAT*α *his3*Δ*1 leu2*Δ*0 lys2*Δ*0 ura3*Δ*0*	ATCC
BY4741-PDE2	*MAT*a *his3*Δ*1 leu2*Δ*0 met15*Δ*0 ura3*Δ*0 pde2Δ::KanMX*	Open Biosystems (Lafayette, CO)
BY4742-RIM15	*MAT*α *his3*Δ*1 leu2*Δ*0 met15*Δ*0 ura3*Δ*0 rim15*Δ*::KanMX*	Ref [50]
BY4742-MSN2	*MAT*α *his3*Δ*1 leu2*Δ*0 lys2*Δ*0 ura3*Δ*0 msn2*Δ*::KanMX*	Ref [50]
BY4742-MSN4	*MAT*α *his3*Δ*1 leu2*Δ*0 lys2*Δ*0 ura3*Δ*0 msn4*Δ*::KanMX*	Ref [50]
BY4742-MSN2/4	*MAT*α *his3*Δ*1 leu2*Δ*0 lys2*Δ*0 ura3*Δ*0 msn2*Δ*msn4*Δ*::KanMX*	Ref [50]
BY4741-Msn2-GFP	*MAT*a *his3*Δ*1 leu2*Δ*0 met15*Δ*0 ura3*Δ*0 Msn2-GFP::HIS3*	Invitrogen (Grand Island, NY)
JB289-1A (TUB1-GFP)	*MAT*a *leu2 his3 ura3::GFP-TUB1:URA3*	Ref [51]
BY4743-BCY1	*MATa/α his3*Δ*1*/*his3*Δ1 *leu2*Δ*0*/*leu2*Δ*0 LYS2*/*lys2*Δ*0 met15*Δ*0*/*MET15 ura3*Δ*0*/*ura3*Δ*0* *bcy1*Δ::*KanMX*/*bcy1*::*KanMX*	Open Biosystems

Triclabendazole, imidazole, benzimidazole, nocodazole, benomyl and albendazole were purchased from Sigma-Aldrich. 2,3-dichlorophenol, thiabendazole, fenbendazole, and mebendazole were purchased from Santa Cruz Biotechnology (Santa Cruz, CA). Triclabendazole sulfone and triclabendazole sulfoxide were purchased from Wako Pure Chemical Industries (Osaka, Japan). The drugs had purities ranging from 98% to 99.5%, based on high performance liquid chromatographic analysis. The drugs were dissolved in dimethyl sulfoxide (DMSO, Sigma-Aldrich) to make 5 to 50 mM stock solutions. cAMP and 2,5-dideoxy-adenosine (2,5-DDA) were purchased from Enzo Life Science (Farmingdale, NY). Unless noted otherwise, all other chemicals were purchased from Sigma-Aldrich.

### Growth, chronological aging and stress resistance assays

For the growth analysis of yeast, cells were inoculated into liquid SC-glucose medium with indicated drug or vehicle (DMSO, 0.1%), incubated at 30°C, and the absorbance (*A*_600 nm_) was monitored over time. Doubling times were determined by the formula: doubling time  =  t/g, where g  =  [log_10_ (*A*_t_/*A*_0_)]/0.3, *A*_0_ and *A*_t_ are absorbance values at time 0 and t, respectively. Values were verified using the online calculator *Doubling Time Software v 1.0.10* (http://www.doubling –time.com).

The chronological aging assay was performed as described in [12], [43]. Yeast cells were pre-grown in liquid YPD medium for 2 days at 30°C to a density of 5–6×10^8^ cells/ml. Cells were then washed and resuspended in the same volume of water, and 10 μl of the culture was inoculated into 5 ml SC-glucose media with or without the drug of interest. Diluted cultures were incubated until stationary phase (48 h) at 30°C, and then the chronological life span assay was started. Viability was determined as described [12], [43]. A survival curve gives the percent of viable cells *versus* time. Each experiment was performed on at least three biological replicate cultures (independently grown from different single colonies on different days).

The stress resistance assay was done using aged cultures. TCBZ (5 μM) was added to diluted cells in liquid culture (SC-glucose), and after 4 days of incubation with shaking at 30°C, cells were removed from culture and serially diluted with phosphate-buffered saline (PBS) buffer and subjected to oxidative stress (100 mM H_2_O_2_ or 300 mM menadione for 60 min) or heat stress (50°C for 60 min). After the stress treatment, cells were spotted onto YPD plates, which were then incubated for 3 days at 30°C.

### Fluorescence microscopy

For experiments with TUB1-GFP and MSN2-GFP strains, cells were inoculated in liquid SC-glucose medium and incubated with shaking at 30°C until mid-log phase. The indicated drug was added and the samples were incubated at 30°C for 1 to 2 h. Cells were washed with PBS and imaged by fluorescence microscopy. For nuclear staining, cells were stained with 0.5 μg/ml DAPI (Sigma-Aldrich), incubated for 10 min at 30°C, washed with PBS and then imaged by fluorescence microscopy. Information on the microscope and image acquisition may be found in [44].

### Trehalose determination

Trehalose was measured using the trehalase-glucose oxidase assay [45]. Yeast cells were inoculated in liquid SC-glucose medium (20 ml) with the indicated drug or DMSO for 1 to 2 d. Samples were centrifuged at 7,000×g for 5 min. Medium was discarded, and suspended in 1 ml ice-cold water. The samples were transferred into microcentrifuge tubes and centrifuged for 30 s at 13,300×g. The supernatant was discarded, and the remaining liquid was completely drained using a syringe connected to vacuum. 0.25 ml of 0.25 M Na_2_CO_3_ solution was added and incubated in water bath set at 95–98°C for 4 h, and then 0.15 ml of 1 M acetic acid and 0.6 ml of 0.2 M sodium acetate were added into the tubes, and gently mixed. Samples were centrifuged for 5 min at 13,300×g, and 500 µl of each sample was removed and incubated with 0.05 U/ml of trehalase overnight at 37°C. Trehalose was degraded to free glucose, and the liberated glucose was measured. The portion of the samples was incubated with glucose oxidase reagent for 30 min at 37°C and absorbance was measured at 420 nm.

### cAMP assay

Cyclic AMP (cAMP) assay was performed using an enzyme immunoassay system (Cyclic AMP XP® Assay kit; Cell Signaling Technology; Danvers, MA) [21], [46]. In yeast, cells (WT and *pde2*Δ; BY4741) were inoculated in SC-glucose medium and incubated at 30°C with shaking until mid-log phase (*A*_600 nm_  = 0.5–0.6), and then indicated drug were added, incubated again for the indicated times. Cells were washed three times with phosphate buffered saline buffer (PBS) (pH 7.4), and were suspended in 1 X cell lysis buffer (200 μl), which is provided by the cAMP assay kit. Cells were homogenized with glass beads (Sigma Aldrich). Supernatants were collected, and cAMP was determined using the cAMP assay kit as indicated by the manufacturer. Protein was determined with Bio-Rad protein assay kit (Hercules, CA).

### Adenylyl cyclase activity

Plasma membrane fractions were prepared [47] and adenylyl cyclase activity was measured by the nonradioactive enzymatic method of Matsumoto et al. [48]. Yeast cells (wild-type BY4741) were inoculated into liquid SC-glucose medium and incubated at 30°C overnight (*A*_600 nm_  = 3.5–4.0). Cells were harvested, washed with 1 M sorbitol containing 20 mM potassium phosphate buffer (pH 7.0) (buffer A) and resuspended in 1 ml of buffer A. Zymolase (2000 units) was added, the mixture was incubated at 30°C for 1.5 h and then 4 ml of chilled buffer A was added. The spheroblasts were collected by centrifugation and gently resuspended in 1 ml of 0.8 M sorbitol solution containing 10 mM MgCl_2_, 1 mM CaCl_2_, 1 mM MnCl_2_, 0.1 mM EDTA, and 50 mM Tris-HCl (pH 7.5) (buffer B). An equal volume of concanavaline A (0.5 mg/ml in buffer B) was added, the mixture was incubated at 30°C for 10 min and the spheroblasts were harvested, lysed by the addition of 5.5 ml of 25 mM PIPES buffer (pH 6.2) (1 mM MnCl_2_, 0.1 mM EDTA, and 1 mM phenylmethylsulfonylfluoride; buffer C), and homogenized in a Dounce glass homogenizer. The crude plasma membrane fraction was collected after centrifugation at 20,000×*g* for 45 min and resuspended in 1 ml buffer C. To solubilize membrane-bound adenylyl cyclase, polyethylene glycol ether W-1 was added to the crude plasma membrane fraction (about 5 mg of protein/ml) to a final concentration of 1% and kept for 60 min at 4°C. This extract was used for adenylyl cyclase activity assay.

For the adenylyl cyclase activity assay, 1.0 µl of 0.1 mM guanosine 5′-[β,γ-imido]triphosphate trisodium salt hydrate (GppNHp) was added to each reaction tube and maintained on ice. GppNHp is an effector that activates adenylyl cyclase [49]. Next, 25 µl of reaction mixture (100 mM Tris-acetate (pH 7.4), 20 mM KCl, 10 mM MgCl_2_, 20 mM phosphoenolpyruvate, 2 mM ATP, 0.02 mM GTP, 2 mM dithiothreitol, 0.04% bovine serum albumin, 0.2 mM theophylline, 1.0 mg/ml pyruvate kinase, and the indicated drug was added to each reaction tube. Finally, 25 µl of the extract was added to each tube and the reaction was initiated by placing tubes in a water bath at 37°C. After 30 min, the reaction was stopped by the addition of 50 µl of 50 mM NaOH, and the samples were heated for 5 min at 95°C. Newly synthesized cAMP was determined using the immunoassay (see above). Adenylyl cyclase activity was reported as pmol cAMP/mg protein/min at 30°C.

### Statistical methods

Estimation of the mean life span (*t*_1/2_) was performed using GraphPad Prism 5.04 (GraphPad Software, San Diego, CA) by fitting a Boltzmann Sigmoidal curve of the survival percentage in individual experiment. ANOVA followed by Tukey's multiple comparison test was used to compare the difference among the groups. Two-way ANOVA was used to test for differences between wild-type and mutants. Statistical software SAS 9.2 for windows was used to perform this analysis (SAS institute Inc. Gary, NC). All *p* values <0.05 were considered statistically significant. Except for mean life span, a Student's t test was used to calculate *p*-values. EXCEL was used for this analysis.

## Supporting Information

Figure S1 **High dose triclabendazole slightly increases mutation frequency (Can resistance).** Cells (wild-type) were inoculated in SC-glucose medium with the triclabendazole or DMSO and incubated at 30°C with shaking. Then, chronological aging experiment was performed as indicated in figure 1B. In order to determine the canavanine-resistance mutants (Can^r^) in the liquid culture, 100 μl aliquot (about 2×10^7^ cells) was harvested from the liquid culture and plated on SC-glucose (without arginine) containing 60 μg/ml L-canavanine sulfate. The mutation frequency was expressed as the ratio of Can^r^ to total viable cells. Values are the mean ± SD of the four independent experiments (n = 3).
(TIF)

Figure S2 **Fenbendazole activates nuclear localization of the Msn2 and decreases the intracellular level of cAMP.** (A) Triclabendazole and fenbendazole. (B) Msn2-GFP localization. Cells expressing Msn2-GFP were inoculated in SC-glucose medium and incubated at 30°C with shaking until mid-log phase, and then indicated drug (5 μM) or DMSO (0.1%) was added. The cells were imaged by fluorescence microscopy after 2 h incubation with the drug. (C) Plot of cells containing nuclear localized Msn2-GFP. Values were obtained from four independent experiments, where the total number of cells counted was 200–250. Error bars are ± SD *, *p*<0.001 (versus DMSO). (D) Intracellular cAMP assay. Cells (wild-type) were inoculated in SC-glucose medium, incubated at 30°C with shaking until mid-log phase, the indicated drug was added, and the samples were incubated for the indicated times. cAMP content was determined with an immunoassay. Values are the mean ± SD of the three independent experiments.
(TIF)

Figure S3 **Bcy1 is required for triclabendazole biological activity.** (A) Doubling time. Cells (wild-type, WT, or *bcy1*Δ/*bcy1*Δ) were inoculated in SC-glucose medium with the indicated drug or DMSO and incubated at 30°C with shaking. Doubling time values are the mean ± SD of the three independent experiments. *, *p*<0.005 (versus WT DMSO). (B) Plot of mean life span from chronological aging assay. *bcy1*Δ/*bcy1*Δ cells were inoculated in SC-glucose medium with triclabendazole or DMSO, incubated at 30°C with shaking for 48 h ( =  zero point of survival curves), and then the aging experiment was started. Life span values (*t_1/2_*) are the mean ± SD. of three independent experiments.
(TIF)

Table S1 **The effects of triclabendazole and related compounds on growth and life span.** Several structural analogs of triclabendazole were evaluated for their effects on growth (doubling time) and survival (t_1/2_) in the chronological life span assay.
(DOC)

Table S2 **The effects of triclabendazole and its metabolites on growth and life span.** Triclabendazole, the triclabendazole sulfoxide (-SO), and the triclabendazole sulfone (-SO_2_) were evaluated for their effects on growth (doubling time) and survival (t_1/2_) in the chronological life span assay.
(DOC)

Text S1 **Materials and methods for the determination of the spontaneous mutation rates of yeast cells as a function of added triclabendazole.**
(DOC)
