# Supplementary figures and images for: The Small Molecule Triclabendazole Decreases the Intracellular Level of Cyclic AMP and Increases Resistance to Stress in Saccharomyces cerevisiae
Source: PLoS One. 2013 May 8;8(5):e64337. doi: 10.1371/journal.pone.0064337 (PMC3648474; doi:10.1371/journal.pone.0064337)

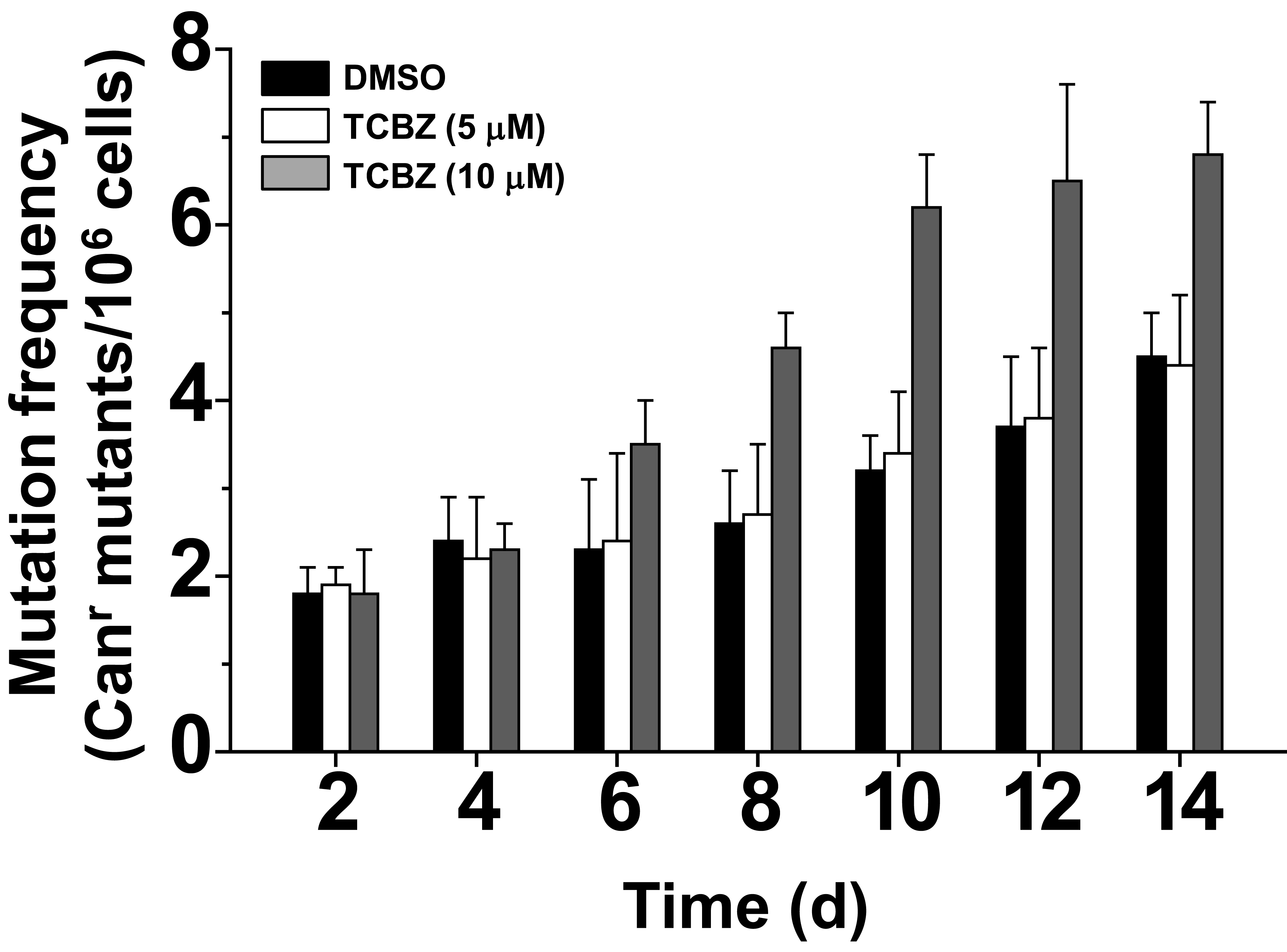

Supplement: Figure S1 — High dose triclabendazole slightly increases mutation frequency (Can resistance). Cells (wild-type) were inoculated in SC-glucose medium with the triclabendazole or DMSO and incubated at 30°C with shaking. Then, chronological aging experiment was performed as indicated in figure 1B. In order to determine the canavanine-resistance mutants (Canr) in the liquid culture, 100 μl aliquot (about 2×10^7^ cells) was harvested from the liquid culture and plated on SC-glucose (without arginine) containing 60 μg/ml L-canavanine sulfate. The mutation frequency was expressed as the ratio of Canr to total viable cells. Values are the mean ± SD of the four independent experiments (n = 3). (TIF) [file pone.0064337.s001.tif]

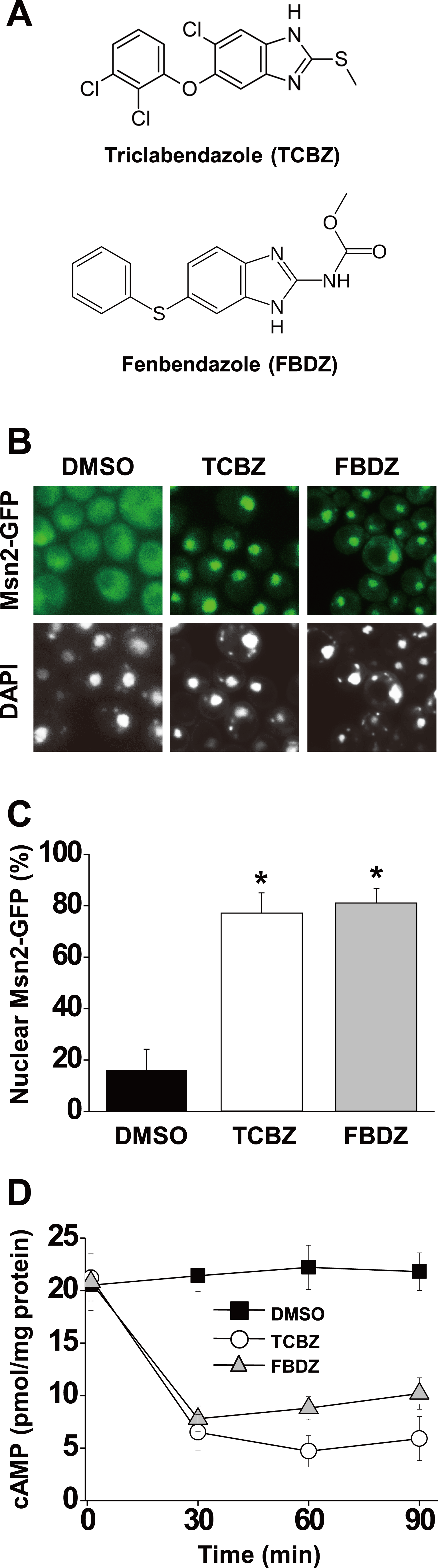

Supplement: Figure S2 — Fenbendazole activates nuclear localization of the Msn2 and decreases the intracellular level of cAMP. (A) Triclabendazole and fenbendazole. (B) Msn2-GFP localization. Cells expressing Msn2-GFP were inoculated in SC-glucose medium and incubated at 30°C with shaking until mid-log phase, and then indicated drug (5 μM) or DMSO (0.1%) was added. The cells were imaged by fluorescence microscopy after 2 h incubation with the drug. (C) Plot of cells containing nuclear localized Msn2-GFP. Values were obtained from four independent experiments, where the total number of cells counted was 200–250. Error bars are ± SD *, p<0.001 (versus DMSO). (D) Intracellular cAMP assay. Cells (wild-type) were inoculated in SC-glucose medium, incubated at 30°C with shaking until mid-log phase, the indicated drug was added, and the samples were incubated for the indicated times. cAMP content was determined with an immunoassay. Values are the mean ± SD of the three independent experiments. (TIF) [file pone.0064337.s002.tif]

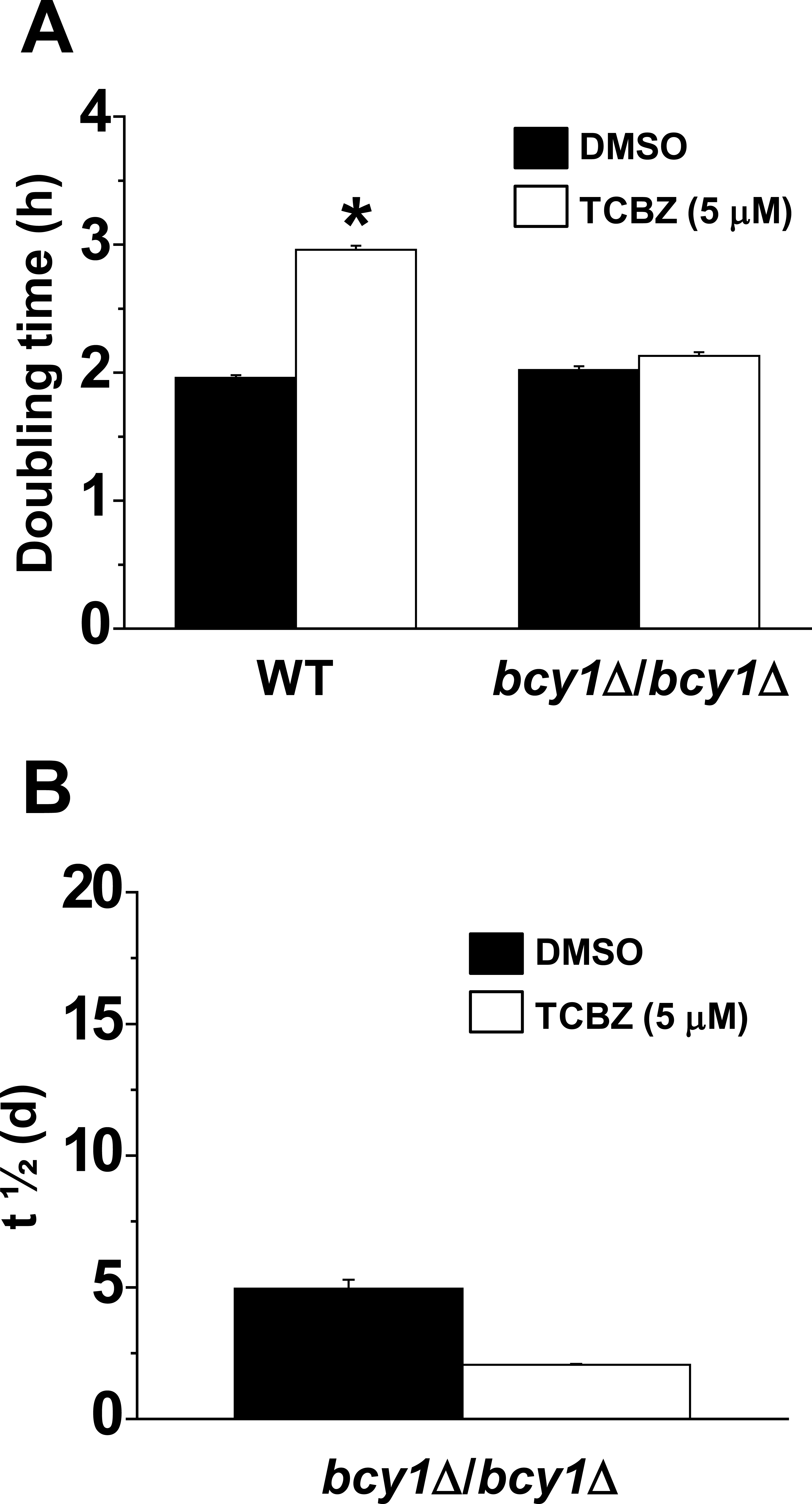

Supplement: Figure S3 — Bcy1 is required for triclabendazole biological activity. (A) Doubling time. Cells (wild-type, WT, or bcy1Δ/bcy1Δ) were inoculated in SC-glucose medium with the indicated drug or DMSO and incubated at 30°C with shaking. Doubling time values are the mean ± SD of the three independent experiments. *, p<0.005 (versus WT DMSO). (B) Plot of mean life span from chronological aging assay. bcy1Δ/bcy1Δ cells were inoculated in SC-glucose medium with triclabendazole or DMSO, incubated at 30°C with shaking for 48 h ( = zero point of survival curves), and then the aging experiment was started. Life span values (t1/2) are the mean ± SD. of three independent experiments. (TIF) [file pone.0064337.s003.tif]
