# Supplementary material for: The Small Molecule Triclabendazole Decreases the Intracellular Level of Cyclic AMP and Increases Resistance to Stress in Saccharomyces cerevisiae
Source: PLoS One. 2013 May 8;8(5):e64337. doi: 10.1371/journal.pone.0064337 (PMC3648474; doi:10.1371/journal.pone.0064337)
Supplement: Table S1 — The effects of triclabendazole and related compounds on growth and life span. Several structural analogs of triclabendazole were evaluated for their effects on growth (doubling time) and survival (t1/2) in the chronological life span assay. (DOC) [file pone.0064337.s004.doc]

**Table S1. The effects of triclabendazole and related compounds on growth and life span.**

| Drug | | Doubling Time (h) | *t*_1/2_ (d) | % change*^a^* | *P* value*^b^* |
| --- | --- | --- | --- | --- | --- |
| DMSO |  | 1.96 ± 0.02 | 8.35 ± 0.12 |  |  |
| Triclabendazole | 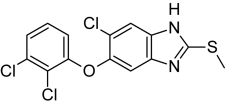 | 2.96 ± 0.05 | 17.2 ± 0.31 | + 106 | < 0.001 |
| Imidazole | 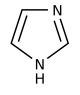 | 1.96 ± 0.02 | 8.50 ± 0.16 | + 1.8 | 0.9998 |
| Benzimidazole | 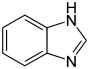 | 1.93 ± 0.03 | 7.95 ± 0.07 | − 4.8 | 0.9214 |
| Dichlorophenol | 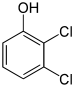 | 1.96 ± 0.02 | 7.57 ± 0.06 | − 9.3 | 0.2541 |
| Albendazole | 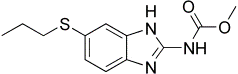 | 1.94 ± 0.03 | 7.96 ± 0.17 | − 4.7 | 0.9272 |
| Thiabendazole | 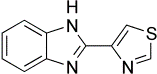 | 1.95 ± 0.02 | 7.99 ± 0.1 | − 4.3 | 0.9566 |
| Fenbendazole | 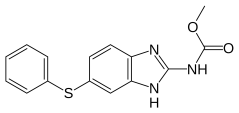 | 3.11 ± 0.04 | 16 ± 0.44 | + 91 | < 0.001 |
| Mebendazole | 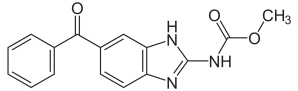 | 1.95 ± 0.01 | 7.84 ± 0.18 | − 6.1 | 0.757 |

*^a^*The percentage change in *t*_1/2_ values was determined versus control cells (DMSO). *^b^*P values for *t*_1/2_ values were determined by an ANOVA analysis with a Tukey post-hoc comparison test of the various samples versus control cells (DMSO).

For the growth analysis, wild-type yeast cells were inoculated in liquid SC-glucose medium with indicated drug or vehicle (DMSO, 0.1%), incubated at 30°C, and the absorbance (*A*_600 nm_) was monitored over time. Doubling times were determined by the formula: doubling time = t/g, where g = [log_10_ (*A*_t_/*A*_0_)] / 0.3, *A*_0_ and *A*_t_ are absorbance values at time 0 and t, respectively. For the mean life span analysis, the survival of a culture of wild-type stationary-phase yeast cells was monitored over several days in liquid SC-glucose media at 30°C. *t*_1/2_ is the time at which 50% of the cells are dead. Values for both experiments are means ± SD of three to four independent experiments.
