# Supplementary material for: The Small Molecule Triclabendazole Decreases the Intracellular Level of Cyclic AMP and Increases Resistance to Stress in Saccharomyces cerevisiae
Source: PLoS One. 2013 May 8;8(5):e64337. doi: 10.1371/journal.pone.0064337 (PMC3648474; doi:10.1371/journal.pone.0064337)
Supplement: Table S2 — The effects of triclabendazole and its metabolites on growth and life span. Triclabendazole, the triclabendazole sulfoxide (-SO), and the triclabendazole sulfone (-SO2) were evaluated for their effects on growth (doubling time) and survival (t1/2) in the chronological life span assay. (DOC) [file pone.0064337.s005.doc]

**Table S2**. **The effects of triclabendazole and its metabolites on growth and life span.**

| Drug | Concentration (µM) | Doubling Time (h) | *t*_1/2_ (d) | % Change*^a^* | *P* Value*^b^* |
| --- | --- | --- | --- | --- | --- |
| DMSO | − | 1.96 ± 0.01 | 8.4 ± 0.1 | − |  |
| Triclabendazole | 5 | 2.95 ± 0.04 | 17.2 ± 0.3 | 105 | < 0.001 |
|  | 20 | NG | ND | − |  |
|  | 50 | NG | ND | − |  |
| Triclabendazole-SO | 5 | 1.98 ± 0.03 | 8.6 ± 0.2 | 2.3 | 0.8456 |
|  | 20 | 2.88 ± 0.05 | 15.9 ± 0.5 | 89 | < 0.001 |
|  | 50 | NG | ND | − |  |
| Triclabendazole-SO_2_ | 5 | 1.95 ± 0.02 | 8.1 ± 0.1 | -3.6 | 0.9789 |
|  | 20 | 2.01 ± 0.02 | 11.5 ± 0.4 | 37 | 0.0033 |
|  | 50 | 3.22 ± 0.04 | 15.1 ± 0.9 | 80 | < 0.001 |

*^a^*The percentage change in *t*_1/2_ values was determined versus control cells (DMSO). *^b^*P values for *t*_1/2_ values were determined by an ANOVA analysis with a Tukey post-hoc comparison test of the various samples versus control cells (DMSO). NG, no growth; ND, not determined.

For the growth analysis, wild-type yeast cells were inoculated in liquid SC-glucose medium with indicated drug or vehicle (DMSO, 0.1%), incubated at 30°C, and the absorbance (*A*_600 nm_) was monitored over time. Doubling times were determined by the formula: doubling time = t/g, where g = [log_10_ (*A*_t_/*A*_0_)] / 0.3, *A*_0_ and *A*_t_ are absorbance values at time 0 and t, respectively. For the mean life span analysis, the survival of a culture of wild-type stationary-phase yeast cells was monitored over several days in liquid SC-glucose media at 30°C. *t*_1/2_ is the time at which 50% of the cells are dead. Values for both experiments are means ± SD of three to four independent experiments.
