# Supplementary material for: The Small Molecule Triclabendazole Decreases the Intracellular Level of Cyclic AMP and Increases Resistance to Stress in Saccharomyces cerevisiae
Source: PLoS One. 2013 May 8;8(5):e64337. doi: 10.1371/journal.pone.0064337 (PMC3648474; doi:10.1371/journal.pone.0064337)
Supplement: Text S1 — Materials and methods for the determination of the spontaneous mutation rates of yeast cells as a function of added triclabendazole. (DOC) [file pone.0064337.s006.doc]

**SUPPORTING INFORMATION**

**The Small Molecule Triclabendazole Decreases the Intracellular Level of Cyclic AMP and Increases Resistance to Stress in *Saccharomyces cerevisiae***

Yong Joo Lee^1^, Runhua Shi^2^, and Stephan N. Witt^1*^

^1^Department of Biochemistry and Molecular Biology, Louisiana State University Health Sciences Center at Shreveport, Shreveport, Louisiana, United States of America

^2^ Feist-Weiller Cancer Center, Louisiana State University Health Sciences Center at Shreveport, Shreveport, Louisiana, United States of America

*Correspondence to Stephan N. Witt (e-mail: switt1@ lsuhsc.edu)

PHONE: 318-675-7891 FAX: 318-675-5180

# **Materials and Methods S1**

**Determination of spontaneous mutation rates**

Spontaneous mutation frequency was determined by measuring the frequency of spontaneous mutations of *CAN1* (YEL063C) . Yeast cells were pre-grown in 4 ml of YPD medium in glass tubes with shaking for 2 days at 30°C to a density of 5-6 × 10^8^ cells/ml. Cells were then washed and resuspended in 4 ml water, and 10 μl of the culture was inoculated into 5 ml SC-glucose media with DMSO or triclabendaole and incubated on a rotating roller drum such that the tubes were vertically tilted ~15° from horizontal and rotating at ~50 rpm to maintain the cells in suspension. The roller drum was positioned within an incubator set at 30°C. Diluted cultures were incubated until stationary phase was reached (48 h), and then the experiment assay was started as the chronological aging assay. To determine total viable cells, aliquots were taken at the indicated times, diluted, and plated on YPD plates. The plates were incubated for 3 days at 30°C, and then colony-forming units (cfu) were counted. To identify the canavanine-resistant mutants (Can^r^), 100 μl aliquot (about 5-6 × 10^7^ cells) was harvested from the liquid culture and plated on SC-glucose (without arginine) containing 60 μg/ml L-canavanine sulfate. Mutant colonies were counted after 3–4 d. The mutation frequency was calculated as the ratio of Can^r^ to total viable cells. Each experiment was performed on three times.

1. Madia F, Wei M, Yuan V, Hu J, Gattazzo C, et al. (2009) Oncogene homologue Sch9 promotes age-dependent mutations by a superoxide and Rev1/Polzeta-dependent mechanism. J Cell Biol 186: 509-523.
